# Formation and characterization of solid fat mimetic based on pea protein isolate/polysaccharide emulsion gels

**DOI:** 10.3389/fnut.2022.1053469

**Published:** 2022-11-10

**Authors:** Wenbo Hou, Jie Long, Yufei Hua, Yeming Chen, Xiangzhen Kong, Caimeng Zhang, Xingfei Li

**Affiliations:** ^1^State Key Laboratory of Food Science and Technology, Jiangnan University, Wuxi, Jiangsu, China; ^2^School of Food Science and Technology, Jiangnan University, Wuxi, Jiangsu, China; ^3^Collaborative Innovation Center of Food Safety and Quality Control in Jiangsu Province, Jiangnan University, Wuxi, Jiangsu, China

**Keywords:** solid fat mimetic, pea protein isolate, polysaccharide, emulsion gels, gelation properties

## Abstract

The emulsion gels that can be used as solid fat replacers were produced with different polysaccharides (κ-carrageenan, κC; high-acyl gellan, HA; konjac glucomannanon, and KGM), pea protein isolate (PPI) and sunflower seed oil. The effect of polysaccharide concentration on the texture, rheological property, microstructure, and water holding capacity of the mixed emulsion gels were investigated. Rheological results showed that the presence of polysaccharides enhanced the hardness, storage modulus and resistance against deformation of emulsion gel, where PPI/κC system exhibited superior hardness with a similar level of pig back fat, due to the self-gelation behavior of κC. CLSM and SEM results showed that the presence of κC, HA, and KGM broke the uniform structure of gel network and formed irregular, threadlike, and oval shaped inclusions respectively, resulting in the broken and coalescence of oil droplets. The α-helix content of emulsion gels decreased, while β-sheet, β-turn and random coils slightly increased due to the unfolding of protein during gel formation. This study may offer a valuable strategy for the development of solid fat mimetic with the characteristics closing to the pig back fat.

## Introduction

In recent years, there has been an increasing demand for diet for several reasons, such as health concerns, environmental issues, and sustainability (1, 2). To manufacture healthier and more environmentally friendly simulated meat products has become a focus in the field of meat and relative products development. For example, pea protein isolates have been used as a substitute for meat protein due to their balanced amino acid composition, good nutritional value and functional properties (3–7). On the other hand, animal fat tissue contains a high amount of saturated fatty acids, which is linked with adverse impacts on human health (8). Therefore, various strategies to replace animal fats by healthier options have attracted people’s attention gradually.

Oleogels and emulsion gels have been considered to be the two main technologies for replacing the fats in meat products. Oleogelation is defined as entrapping the liquid oil in a thermo-reversible and three-dimensional gel network using one or more oleogelator agents, and is characterized as semi-solid systems without changing its chemical composition (9, 10). Carnauba wax (CW) based oleogel was reinforced with adipic acid (AA) and it was applied to model cake and beef hamburger. The results showed that the texture and sensory properties of formulated cake and burgers prepared with CW2%/AA4% oleogel were reached the similar level of un-substituted products essentially (11). Moreover, the pork fat in more compact and lighter Bologna sausages was also replaced by monoglyceride oleogels made from traditional or high oleic sunflower oils. This study indicated that a reduction of 50% of pork fat back would not significantly change the hardness of sausages (12). However, it was worth noticing that, the stability of oleogels decreased over time, which was attributed to large crystals in oleogels and less contact points among them. Regarding emulsion gels, the emulsified fats were filled in to protein network, and then gelatinized emulsions to give them solid like mechanical properties (13). Using succinylated chicken liver protein and pre-emulsified sunflower oil to substitute back-fat in emulsified sausages obtained similar texture properties to high back-fat sausages, and also improved the quality and nutritional characteristic of sausages (14). However, replacing animal fats with inulin-based emulsion gels would result in a decrease in hardness of products (15, 16). In order to improve the texture characteristics of fat mimics, some studies had proposed to prepare substitution with fully hydrogenated canola oil and soy protein (17). Through this method, the texture of fat mimetic could be improved, but what was worth thinking was that hydrogenation forms trans-fatty acids had negative impact on human health by decreasing the proportion of “good cholesterol” and increasing the proportion of “bad cholesterol” (18).

Considering the instability of oleogel and the hazards of trans-fatty acids in hydrogenated oils, the objective of this research was to establish a method for preparing solid fat mimetic with higher-protein and lower-fat content by a healthier and environmentally friendly way. Three functional polysaccharides, κ-carrageenan, high-acyl gellan, and konjac glucomannanon have been commonly used as thickener in the food industry, especially for colloid snacks in China. The mixture of polysaccharides and pea protein isolate (PPI) were crosslinked by transglutaminase (TG) to obtain a solid fat mimetic with excellent mechanical property similar to that of pig back fat. The possible influencing rule of solid fat mimetic was investigated from the rheological properties, microstructures, structure information, etc.

## Materials and methods

### Materials

κ-carrageenan (κC, MW∼7313 kDa, detected by HPSEC-MALLS) was purchased from Aladdin Chemistry Co., Ltd., (Shanghai, China). High-acyl gellan (HA, MW∼1435 kDa, detected by HPSEC-MALLS) and konjac glucomannan (KGM, MW∼34 kDa, detected by HPSEC-MALLS) were purchased from Yuanye biological technology Co., Ltd., (Shanghai, China). Sunflower seed oil (SFO) and pig back fat were obtained from a local supermarket. Glutamine transaminases (TG, 100 U/g) were obtained from Beijing Solarbio Science and Technology Co., Ltd., (Beijing, China). Fluorescein isothiocyanate (FITC) and Nile red were obtained from Sigma-Aldrich (St. Louis, MO, USA). All other chemicals were analytical grade and did not require further purification.

### Preparation of pea protein isolate

The PPI was prepared according to the method of Shen et al. (19). Briefly, the alcohol-washed pea meal was mixed with deionized water (1:10, w/v) and added 2.0 M NaOH to adjust the pH to 9.0. After centrifugation at 9,000 rpm, the supernatant was adjusted to pH 4.5 with 1.0 mol/L HCl and then centrifuged again to collect the protein curd. The protein curd was re-dissolved in water by adjusting pH to 7 using 1.0 mol/L NaOH, followed by centrifugation to remove insoluble residues. The above protein solution was freeze-dried and stored at −20°C before the further experiment.

### Preparation of emulsion gels as solid fat mimetic

Polysaccharides (κC, HA, and KGM) were blended with the PPI dispersion to achieve a mixture solution containing 20% (w/w) of protein and 0.2, 0.6, 1.0% (w/w) of polysaccharide, separately. After adjusting to pH 7, the mixture solution was mixed with sunflower seed oil (30%, w/w) and homogenized with a Model V2700 homogenizer (Qingdao Huwazi Electric Appliance Co., Qingdao, China) for 4 min at 22,000 rpm to prepare emulsions. In order to induce gel formation by protein crosslinking, the TG (20 U/g protein) was added to the emulsions. Samples were subsequently incubated at 37°C for 60 min, and then heated at 85°C for 15 min to inactivate the enzyme. Finally, the prepared emulsion gels were stored overnight at 4°C before analysis. The control group in this study was PPI emulsion crosslinked by TG without addition of polysaccharides under the same conditions.

### Texture profile analysis

The textural parameters of emulsion gels and pure pork back fat were determined using TA-XT Plus texture analyzer (Stable Micro Systems Ltd., Godalming, UK) with a P36R probe, according to the method of Lu et al. (14) with some modification. Before experiment, the pre-cooling pig back fat was kept at 25°C for at least 2 h to recover the nature state. The cylinder samples (1.2 cm high, 1.2 cm diameter) were performed with a deformation level of 50% at 25°C using the following parameters: pretest speed, test speed and posttest speed were 2.0, 1.0, and 1.0 mm/s respectively, and contact force was 5 *g*. The four textural parameters hardness, springiness, cohesiveness, and chewiness were recorded in test.

### Rheological measurements

Monitoring the rheological behavior of samples using an oscillatory rheometer (MCR 301, Anton Paar, Graz, Austria) with a parallel plate (PP50, diameter = 50 mm) and 1.0 mm gap. Low-density silicone oil was used to the edge of the parallel plate to prevent the evaporation of the liquid.

#### Temperature sweep and frequency sweep

The strain (0.1%) applied was within the linear viscoelastic region and the oscillation frequency was 0.1 Hz. The emulsions containing TG were heated from 25 to 37°C at a rate of 6°C/min and kept at 37°C for 60 min, then heated to 85°C at 6°C/min and kept at 85°C for 15 min to destroy the enzyme, finally, cooled to 25°C at a cooling rate of 6°C/min. After the temperature cycle, frequency sweeps of gelled simples were carried out in a range of angular frequencies between 6.28 and 126 rad/s with 0.1% strain at 25°C. The power law model was used to characterize the relationship between the G’ and frequency (ω) as follows [Eq. (1)]:


(1) G=′Kωn′′

where K′ is the power law model constant and n′ is the frequency exponent.

#### Creep and recovery tests

Creep-recovery test was executed by applying a constant shear stress, σ_0_, of 50 Pa to the gelled simples for 300.1 s, after which the stress was released, a partial recovery was monitored for a further 600.3 s. Burger’s model was used to interpret the creep data, which was used to characterize the viscoelastic properties. The model was expressed as Eq.(2) (20):


(2) $$J\,\left(t\right)=\frac{1}{{\text{G}}_{0}}+\frac{1}{{\text{G}}_{1}}\,\left(1-{\text{e}}^{-\frac{\text{t}}{\lambda }}\right)+\frac{\text{t}}{\mu_{0}}$$

where J is the creep compliance (1/Pa) which is the ratio of strain to stress; G_0_ and G_1_ respectively represent the instantaneous elastic modulus (Pa) and retarded elastic modulus (Pa); λ represents the retardation time of Kelvin component (s), and μ_0_ is the viscous part (Pa s^–1^) of Newtonian element.

### Microstructure characterization

#### Confocal laser scanning microscopy

The protein phase was stained with fluorescein-5-isothiocyanate (0.1%, w/v) and the lipid phase was labeled by Nile Red (0.1%, w/v) prior to adding the TG dispersion. Afterward, the emulsion gels were prepared as described above, and imaged with a confocal laser scanning microscope (LSM710 Carl Zeiss AG Germany) objective. For locating the PPI and the oil droplets, FITC and Nile red were excited at 514 and 488 nm respectively.

#### Scanning electron microscopy

The liquid nitrogen pre-frozen samples were firstly freeze-dried to fix their structures, and then the oil phase was removed according to the method of Li et al. (21) with some alterations. Briefly, the cut sheet samples were soaked in petroleum ether for 24 h and this procedure was repeated three times, then the defatted samples were placed in a vacuum drying oven at 50°C for 4 h to evaporate the petroleum ether. A SEM QUANTA 200 (FEI Company, Hillsboro, OR, USA) was used to observe the microstructure of samples. The acceleration voltage was 10.0 kV, and the microtopography of the samples was observed at 100× and 1000×, respectively.

### Low field nuclear magnetic resonance

Low field nuclear magnetic resonance (LF-NMR) relaxation tests were carried out with a LF-NMR analyzer (MesoMR23-060V-I, Niumag Analytical Instruments, Shanghai, China) to evaluate the state and distribution of water in samples. Approximately 3 g of gel was placed into a cylindrical glass tube and the T_2_ relaxation time was measured using the CPMG sequence. The parameters were as follows: echo time, 0.3 ms; radio frequency delay time, 0.08 ms; waiting time, 3,500 ms and the number of scans, 8. A total of 15,000 echoes were acquired for analysis.

### Determination of water holding capacity

The water holding capacity (WHC) was analyzed by a centrifugal procedure described by Qayum et al. (22) with some alterations. A certain amount of the sample was transferred to centrifuge tubes and centrifuged at 10,000 g at 4°C for 15 min, then refused the supernatant. WHC was defined as the percentage of sample weight after centrifugation (W_2_) to its pre-centrifugation weight (W_1_). The WHC was calculated using the following Eq. (3):


(3) $$\text{WHC}\left(\%\right)=\frac{W_{2}}{W_{1}}\times 100$$

### FTIR spectroscopy

Infrared spectra were recorded using a Thermo Nicolet Nexus 470 FTIR spectrometer (Thermo Nicolet Analytical Instruments, MA, USA). The freeze-dried and crushed samples were mixed with potassium bromide and pressed into tablets for further FTIR measurement. The infrared spectrum had a scanning range of 4,000 to 400 cm^–1^, a resolution of 4 cm^–1^ and 32 scanning times. The spectrum results were determined and computed by OMNIC (Ver.8.2) and Peakfit (Ver.4.12).

### Statistical analysis

All tests were repeated at least triplicated. Statistical analysis was performed using SPSS 26.0 software (SPSS Inc., Chicago, IL, USA). In order to test the significant differences of results between different groups, Duncan test was used for one-way analysis of variance (ANOVA). Differences were considered significant at *p* < 0.05.

## Results and discussion

### Textural properties

Table 1 illustrates the hardness, springiness, cohesiveness and chewiness of the emulsion gels containing different concentration of κC, HA, and KGM. Hardness was one of the important texture parameters to evaluate the quality of gels and chewiness was the simulated energy when a sample was chewed to the point where it can be swallowed. The increase of hardness means more energy was demanded for chewing (23). Both the hardness and chewiness of the PPI/polysaccharide emulsion gels were higher than that of PPI emulsion gel (0% group). With the increase of polysaccharide concentration, the hardness and chewiness showed an increasing trend for all three types of polysaccharides. At polysaccharide concentration of 1.0%, the maximum hardness of PPI/κC, PPI/HA, and PPI/KGM emulsion gels reached a level of 1951.49, 1792.91, and 1669.91 g respectively; and the hardness of PPI/κC emulsion gel showed the very similar level to that of pig back fat. A steric exclusion mechanism was reported to be related to the gel hardness and chewiness of protein/polysaccharide mixed gels containing no oil phase (24). In the mixed emulsion gel system, the increase of the polysaccharide concentration promoted the mutual attraction between protein molecules and reduced the contact area of the protein with the surrounding solution and the filled oil droplets might further enhanced the steric exclusion. In addition, protein and polysaccharide were thermodynamically incompatible at neutral pH (25, 26), which can result in microphase separation, and thus leading to an increase in regional effective protein concentration and protein–protein interactions during the gel formation. The gel hardness of PPI/κC emulsion gel was visibly higher than that of PPI/HA and PPI/KGM gels, which might be attributed to the synergistic interaction between κC self-gel and PPI (see section “Rheological behaviors”), because gelation behavior of HA and KGM was weak at the very low ionic strength and neutral pH (27, 28).

**TABLE 1:** The texture profile analysis (TPA) data of emulsion gels containing different polysaccharides.

Sample	Hardness (g)	Springiness (%)	Cohesiveness	Chewiness
0%	1389.12 ± 39.98^d^	96.75 ± 2.35^a^	0.91 ± 0.01^a^	1224.24 ± 49.21^c^
0.2% κC	1569.69 ± 48.17^c^	95.19 ± 1.38^ab^	0.89 ± 0.01^b^	1337.42 ± 28.37^b^
0.6% κC	1790.86 ± 69.03^b^	93.45 ± 2.18^b^	0.88 ± 0.02^bc^	1478.53 ± 140.69^a^
1.0% κC	1951.49 ± 63.85^a^	93.47 ± 1.35^b^	0.87 ± 0.01^c^	1581.78 ± 88.22^a^
0%	1389.18 ± 39.98^d^	96.75 ± 2.35^a^	0.91 ± 0.01^a^	1224.24 ± 49.21^c^
0.2% HA	1471.84 ± 35.70^c^	95.39 ± 0.61^ab^	0.90 ± 0.00^b^	1356.05 ± 52.00^b^
0.6% HA	1581.40 ± 72.00^b^	95.04 ± 0.56^b^	0.89 ± 0.00^c^	1371.52 ± 36.19^b^
1.0% HA	1792.91 ± 75.20^a^	94.69 ± 0.66^b^	0.89 ± 0.01^d^	1506.44 ± 57.63^a^
0%	1389.18 ± 39.98^c^	96.75 ± 2.35^a^	0.91 ± 0.01^a^	1224.24 ± 49.21^c^
0.2% KGM	1431.51 ± 37.99^c^	96.71 ± 1.32^a^	0.91 ± 0.00^a^	1262.75 ± 30.71^c^
0.6% KGM	1545.30 ± 80.85^b^	95.97 ± 1.57^a^	0.91 ± 0.00^a^	1363.71 ± 77.67^b^
1.0% KGM	1669.91 ± 69.78^a^	95.77 ± 1.21^a^	0.90 ± 0.00^b^	1466.63 ± 66.60^a^
PBF	1971.56 ± 134.22	69.49 ± 1.65	0.69 ± 0.02	954.84 ± 89.54

Different letters represent significant differences between different samples (*P* < 0.05).

κC, κ-carrageenan; HA, high-acyl gellan; KGM, konjac glucomannanon; PBF, pig back fat.

The springiness indicated how well samples could return to their original state after being compressed. On the other hand, cohesiveness was a standard to measure the deformation resistance of gel. The smaller the cohesiveness, the greater the damage to the irreversible structure after compression (17). The springiness and cohesiveness decreased with the increase in the content of polysaccharides, but still higher than that of pig back fat. The higher chewiness of the mixed gels also reflected an elastic dominated gel. This could be due to the limited content of polysaccharides that cannot affect protein dominated gelation behavior in mixed gels.

### Rheological behaviors

**FIGURE 1:**
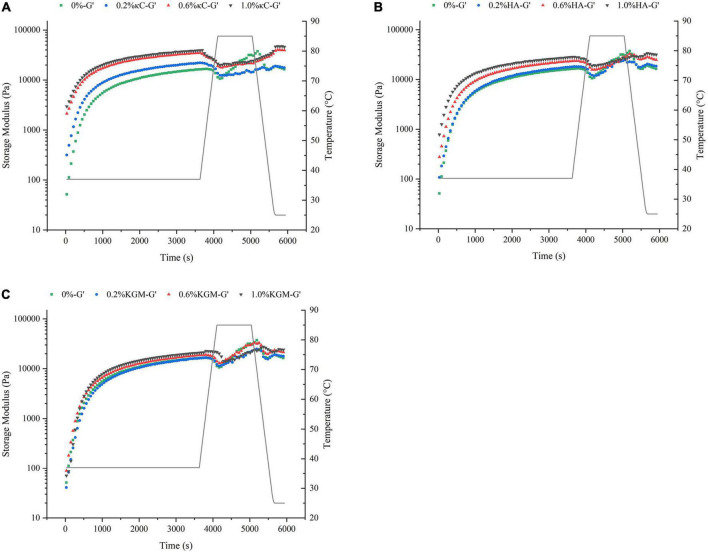
Temperature cycling of the storage modulus (G’) of emulsion gels containing different polysaccharides: **(A)** κC, **(B)** HA, and **(C)** KGM. κC, κ-carrageenan; HA, high-acyl gellan; KGM, konjac glucomannanon.

**FIGURE 2:**
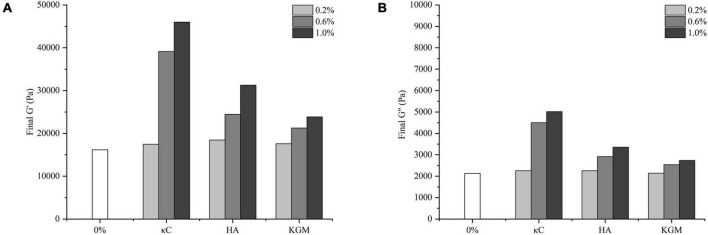
Final G’ **(A)** and G” **(B)** of the emulsion gels containing different polysaccharides. κC, κ-carrageenan; HA, high-acyl gellan; KGM, konjac glucomannanon.

The storage modulus (G’) reflected the contribution of the elastic portion of gels and characterized the strength of gels. The changes of G’ of the emulsion gels containing 0.2–1.0% (w/w) κC, HA and KGM subjected to the temperature cycling were shown in Figure 1. The G’ of all emulsion gels exhibited a sharp increase in 30 min at the first insulation stage (37°C), leveled off gradually with the increase in insulation time. The G’ values of emulsion gels for three κC, HA and KGM systems at this stage were higher than that of pure PPI emulsion gel, and gradually increased as the increase in polysaccharide concentration, which confirmed the enhanced hardness of PPI/polysaccharide emulsion gels. At the second insulation stage (85°C) and the followed cooling stage, the gelation curves of G’ of the PPI/polysaccharide emulsion gels became different compared to pure PPI emulsion gel depending on the types and concentration of polysaccharide. For PPI/κC emulsion gels, the gel strength of G’ increased more slowly than that of pure PPI emulsion gel and formed a “kink” in the curve of G’. This “kink” curve of G’ has been reported in the BSA/carrageenan mixed gels, which was considered as the result of melting and gelation behavior of carrageenan (29). It can be inferred that the heat-induced melting and colling gelation behavior of κC helps to improve the final gel strength. The G’ curves of PPI/KGM emulsion gel almost followed the very similar path of pure PPI emulsion gel at the second insulation stage (85°C) and the followed cooling stage. The reason is that KGM had little gelation behavior at low concentration, showing little impact on the protein gelation behavior (25). The G’ curves of PPI/HA emulsion gel was similar to that of pure PPI emulsion gel at low concentration (0.2%), and showed slight kinks at 0.6 and 1.0%, suggesting that the gelation behavior of PPI became stronger at high HA content due to the non-Newtonian property of HA over 0.6% (30). Meanwhile, the incorporation of polysaccharides also improved the effective concentration of PPI by the steric exclusion (24), altering the gel formation process. The different rheological behavior of κC, HA and KGM in emulsion gels also affected the final values of G’ and G” after the temperature cycling, as shown in Figure 2. It was found that the final G’ and G” values of the emulsion gels varied in decreasing order of PPI/κC>PPI/HA>PPI/KGM at the same polysaccharide concentration, which were higher than that of pure protein emulsion gel, indicating a maximum synergistic effect of κC in enhancing protein gel strength.

**FIGURE 3:**
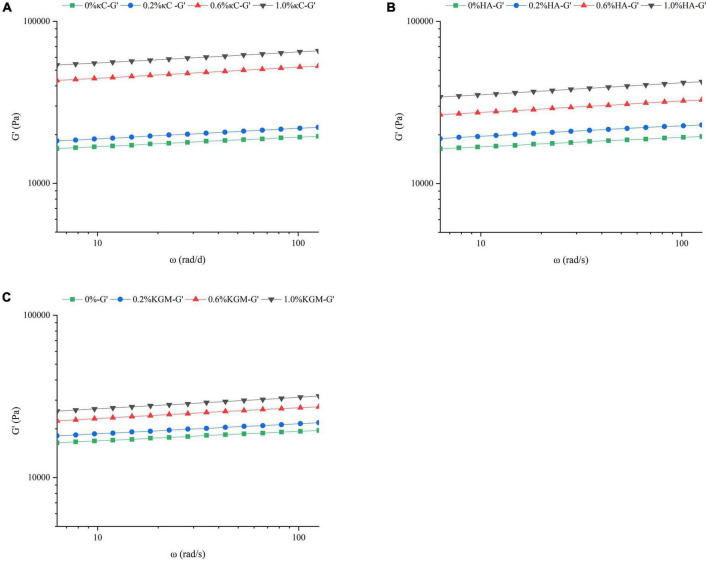
Frequency sweep of the storage modulus (G’) of emulsion gels containing different polysaccharides (**A**, κC, **B**, HA, and **C**, KGM) at 25°C. κC, κ-carrageenan; HA, high-acyl gellan; KGM, konjac glucomannanon.

To further examine the effect of κC, HA, and KGM on the rheological behaviors of emulsion gel system, frequency sweeps, and creep/recovery tests were carried out on the samples. Frequency sweeps provided the mechanical spectrum called the “fingerprint” of gels. Therefore, it was possible to determine the effect of the oscillatory stress rate on G’ on the short time scales of the linear viscoelastic region (LVER). The changes of G’ of the emulsion gels containing κC, HA, and KGM over the frequency range of 6.28–128 rad/s were shown in Figure 3. The addition of different polysaccharides had different degrees of promotion effect on G’. Moreover, G’ exhibited little dependence on the applied frequency suggesting that the network structure of gel was stable and not easy to destroy. The G’ values of PPI/κC emulsion gel at 0.6 and 1.0% polysaccharide were significantly higher than that of HA and KGM systems over the whole frequency range. This phenomenon also corresponded well to the TPA test results in which the PPI/κC emulsion gel had the greatest hardness. Table 2 showed the power law constant (K’) and exponent (n’) derived from the power law model. The values of K’ and n’ increased with the increase in the polysaccharide content, and reached the maximum at 1.0%. The increased K’ of emulsion gels reflected a stronger gel network with higher rigidity (31). The values of n’ of all emulsion gels were low, confirming that the time stability of the covalent bonds (TG induced) production in the gel network (32). Relevant researches showed that the n’ of fully covalently crosslinked gels was 0, while the n’ of physical gels was positive (33). The n’ value increased with the increase of polysaccharide content, suggesting that the non-covalent interactions occurred between protein and polysaccharides.

**TABLE 2:** The parameters of power law function model of emulsion gels containing different polysaccharides at 25°C.

Sample	K’ (10^3^Pa)	n’	*r* ^2^
0% κC	14.73 ± 2.85^d^	0.059 ± 0.001^d^	0.9989
0.2% κC	16.21 ± 1.96^c^	0.065 ± 0.000^c^	0.9997
0.6% κC	37.99 ± 3.19^b^	0.069 ± 0.000^a^	0.9999
1.0% κC	47.36 ± 6.78^a^	0.068 ± 0.000^b^	0.9996
0% HA	14.73 ± 2.85^d^	0.059 ± 0.001^d^	0.9989
0.2% HA	16.87 ± 4.83^c^	0.065 ± 0.001^c^	0.9980
0.6% HA	23.34 ± 2.69^b^	0.071 ± 0.000^b^	0.9997
1.0% HA	29.83 ± 2.8^a^	0.073 ± 0.000^a^	0.9998
0% KGM	14.73 ± 2.85^d^	0.059 ± 0.001^d^	0.9989
0.2% KGM	16.14 ± 2.21^c^	0.062 ± 0.000^c^	0.9995
0.6% KGM	19.87 ± 4.07^b^	0.066 ± 0.001^b^	0.9990
1.0% KGM	22.56 ± 2.41^a^	0.071 ± 0.000^a^	0.9998

Different letters represent significant differences between different samples (*P* < 0.05).

κC, κ-carrageenan; HA, high-acyl gellan; KGM, konjac glucomannanon.

**FIGURE 4:**
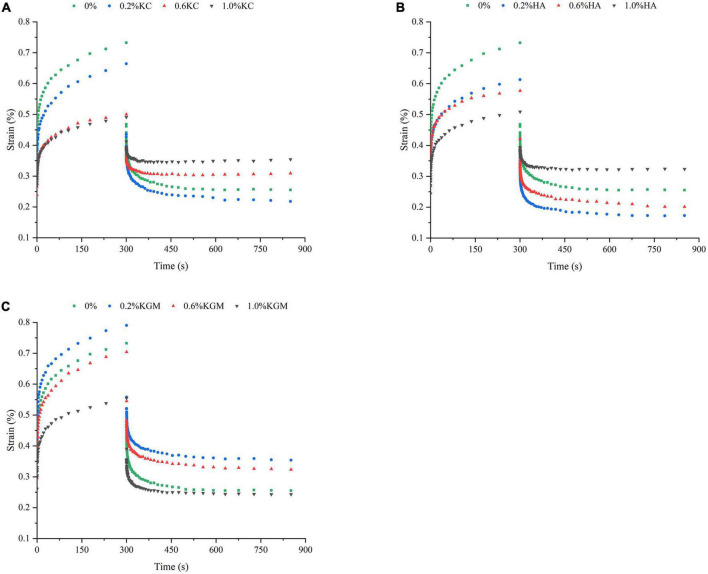
Creep-recovery behavior of emulsion gels containing different polysaccharides: **(A)** κC, **(B)** HA, and **(C)** KGM. κC, κ-carrageenan; HA, high-acyl gellan; KGM, konjac glucomannanon.

**FIGURE 5:**
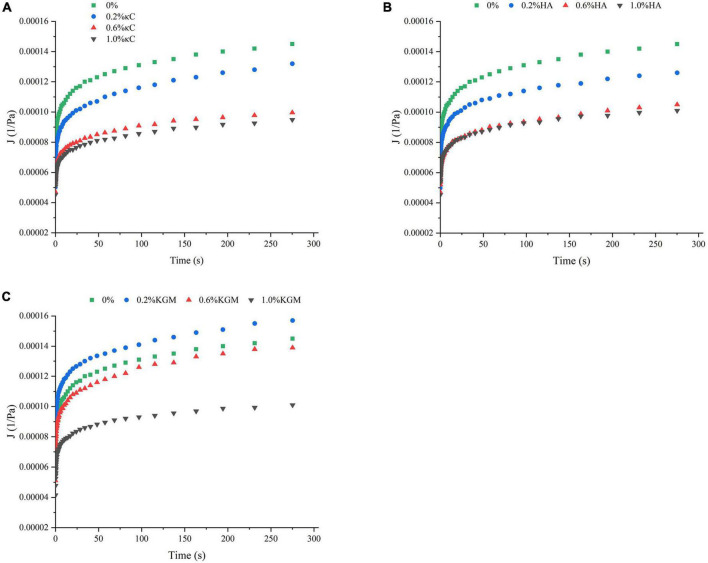
Compliance curve of emulsion gels containing different polysaccharides: **(A)** κC, **(B)** HA, and **(C)** KGM. κC, κ-carrageenan; HA, high-acyl gellan; KGM, konjac glucomannanon.

The rheological properties of emulsion gels at 25°C were also characterized by transient tests, which made it possible to differentiate the structural characteristics of gels on a longer time scale (34). The changes in strain versus time (Figure 4) showed that the deformation degree of pure PPI emulsion gels was greatest, and it became smaller with the increase in the polysaccharide content, the emulsion gels containing 1.0% (w/w) of polysaccharide are least deformed, indicating the high resistance against external force. At 1.0% polysaccharide, the total strain of the emulsion gels increased in the order of PPI/κC<PPI/HA<PPI/KGM, suggesting the stronger gel network of PPI/κC emulsion gel. It was interesting that, the strains of recovery phase of PPI/κC, PPI/HA systems were obviously higher at 0.6 and 1.0% than at 0.2%, while PPI/KGM system showed the opposite trend, suggesting that the gelation of κC and HA reduced the anti-deformability of emulsion gels. Relationship between creep compliance and time could also reflect the rigidly of gels (35). A higher creep compliance (J) value signified a weaker structure, while a lower value indicated a stronger one (31). The creep behavior of emulsion gels was shown in Figure 5. The J of emulsion gels gradually decreased with the increase of polysaccharides content. In Table 3, values of parameters obtained from fitting creep data to Burger’s model (Eq. 2) were listed. It can be seen that the addition of polysaccharides promoted the instantaneous elastic behavior (G_0_) and delayed elastic behavior (G_1_), and they reached the maximum at 1.0% κC, which demonstrated the incorporation of polysaccharides enhanced the structural strength of gels (31). The increase in the μ_0_ value indicated an increase in the viscous component of gels. The results showed that low content of polysaccharides had no noticeable effect on the viscosity, while polysaccharides at high concentrations had a promoting effect, especially κC. For the retardation time of Kelvin component (λ), PPI/κC emulsion gel with high λ values reached complete deformation more slowly than that of PPI/HA and PPI/KGM gels. The creep/recovery results reflected the enhancement of the gel structure in the presence of polysaccharide, and κC had the most significant promotion effect.

**TABLE 3:** The parameters of Burger’s model of emulsion gels containing different polysaccharides.

Sample	G_0_ (10^3^Pa)	G_1_ (10^3^Pa)	λ (s)	μ_0_ (10^6^Pas)	*r* ^2^
0% κC	13.67 ± 0.42^d^	25.09 ± 0.70^d^	3.56 ± 0.03^d^	7.33 ± 0.01^c^	0.9618
0.2% κC	15.2 ± 0.14^c^	30.42 ± 0.84^c^	3.93 ± 0.04^c^	7.30 ± 0.01^c^	0.9670
0.6% κC	17.57 ± 0.31^b^	44.26 ± 0.92^b^	4.71 ± 0.05^b^	11.78 ± 0.02^b^	0.9695
1.0% κC	18.42 ± 0.56^a^	46.38 ± 1.04^a^	4.86 ± 0.05^a^	12.75 ± 0.02^a^	0.9725
0% HA	13.67 ± 0.72^c^	25.09 ± 0.7^d^	3.56 ± 0.03^b^	7.33 ± 0.01^d^	0.9618
0.2% HA	15.40 ± 0.57^b^	29.43 ± 0.82^c^	3.39 ± 0.03^c^	8.57 ± 0.01^c^	0.9614
0.6% HA	17.80 ± 0.56^a^	39.21 ± 0.42^b^	3.99 ± 0.04^a^	10.21 ± 0.02^b^	0.9737
1.0% HA	17.66 ± 0.70^a^	40.21 ± 0.56^a^	3.99 ± 0.04^a^	11.88 ± 0.02^a^	0.9641
0% KGM	13.67 ± 0.42^c^	25.09 ± 0.70^c^	3.56 ± 0.03^a^	7.33 ± 0.01^b^	0.9618
0.2% KGM	12.04 ± 0.42^d^	24.74 ± 0.70^c^	3.51 ± 0.03^b^	7.07 ± 0.01^c^	0.9632
0.6% KGM	14.39 ± 0.24^b^	27.62 ± 0.68^b^	3.03 ± 0.03^d^	6.80 ± 0.01^d^	0.9550
1.0% KGM	18.40 ± 0.29^a^	36.76 ± 0.61^a^	3.20 ± 0.03^c^	11.60 ± 0.02^a^	0.9578

Different letters represent significant differences between different samples (*P* < 0.05). κC, κ-carrageenan; HA, high-acyl gellan; KGM, konjac glucomannanon.

### Microstructure analysis

**FIGURE 6:**
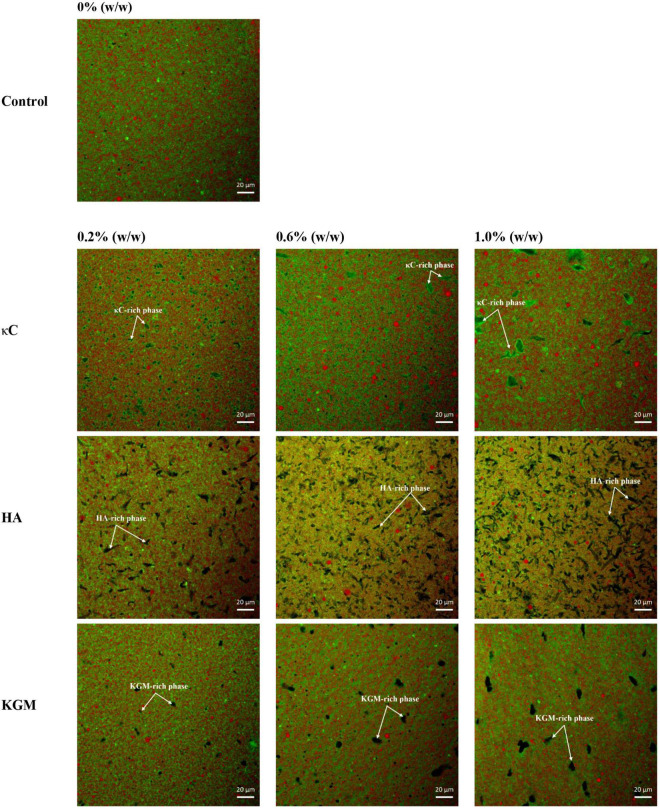
Confocal laser scanning microscopy (CLSM) images of emulsion gels containing different polysaccharides. Scale bar is 20 μm. κC, κ-carrageenan; HA, high-acyl gellan; KGM, konjac glucomannanon.

The microscopic structures of the emulsion gels containing κC, HA, and KGM were characterized by CLSM and SEM. Figure 6 showed the CLSM micrographs of the emulsion gels varied with polysaccharide proportion. The network structures of the emulsion gels without polysaccharide were continuous uninterrupted and the oil droplets were distributed uniformly. Images of emulsion gels containing 0.2% (w/w) κC showed a distinct κC phase which appeared as melanocratic and small inclusions with irregular shapes (as indicated by the arrow in the figures), suggesting the formation of phase separation in PPI/κC emulsion gel. Increasing the concentration of κC to 0.6 or 1.0% further promoted the phase separation behavior with increased size of the κC inclusions. This phenomenon also occurred in PPI/HA and PPI/KGM emulsion gels where the HA-rich phase showed irregular threadlike inclusions and the KGM-rich phase appeared as oval inclusions (as indicated by arrows in the figures). Tobin et al. (25) showed a similar result for whey protein/KGM systems, finding a protein-enriched phase with entrapped KGM inclusions. The formation of this microstructure might be attributed to the polysaccharide-induced microphase separation during gel formation due to thermodynamically incompatibility between two biopolymers. The effective concentration of both polymers increased during phase separation, confining them to a portion of the total volume, thus improving the gel strength of the integral system (25, 36). In addition, compared with homogeneous distribution of oil droplets of the pure protein emulsion gel, the oil droplets of all given mixed emulsion gels became unevenly distributed by forming large and small oil droplets, suggesting polysaccharide induced microphase separation also caused the broken and coalescence of oil droplets.

**FIGURE 7:**
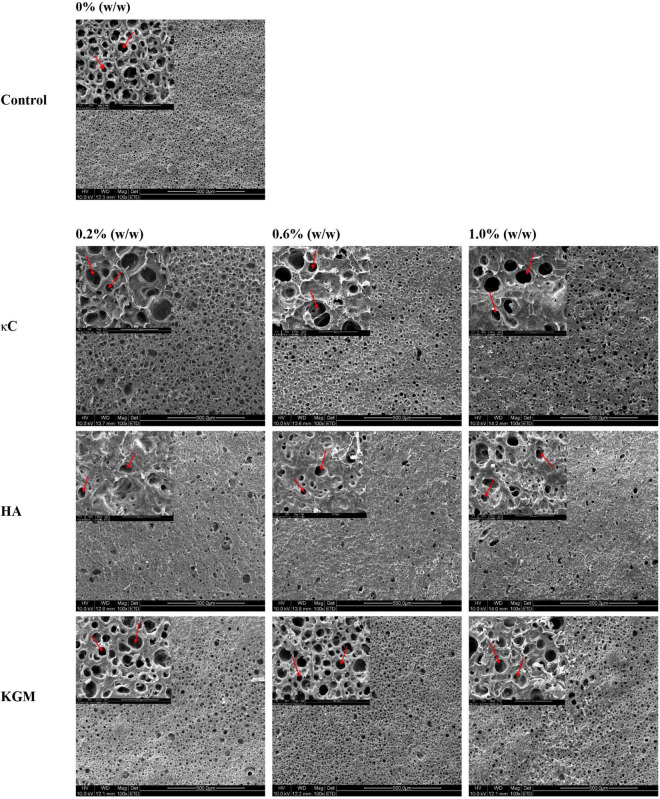
Scanning electron microscopy (SEM) images (100×) of emulsion gels containing different polysaccharides. The holes represent oil droplets; the insert figure represents the details of emulsion gel at (1000×). κC, κ-carrageenan; HA, high-acyl gellan; KGM, konjac glucomannanon.

Further investigation of the evolution of emulsion gel microstructure was conducted using SEM. As shown in Figure 7, the coarser and less homogenous structures were formed as the polysaccharide concentration increased. The 1000× pictures provided more details of the emulsion gels (inset). As shown in the images, a homogeneous structure that consisted of protein networks and oil droplets (circular shaped holes) with uniform size was observed in the PPI emulsion gels. As the concentration of polysaccharides increased, the structures of the PPI/polysaccharide emulsion gels became more heterogeneous with oil droplets of different sizes (see inset). Compared to PPI emulsion gels, the oil droplets in PPI/HA emulsion gels became larger in size, while the oil droplets for PPI/κC emulsion gels became smaller, due to the presence of a large amounts of irregular inclusions causing the broken of oil droplets (Figure 6, HA image). Combined with CLSM images, it seemed that HA-rich phase was disordered in the gels, while KGM-rich phase was evenly distributed in the gels. The difference in the gel microstructure among different polysaccharides further affected the textural and rheological properties of the three emulsion gel systems.

### Water holding properties

**FIGURE 8:**
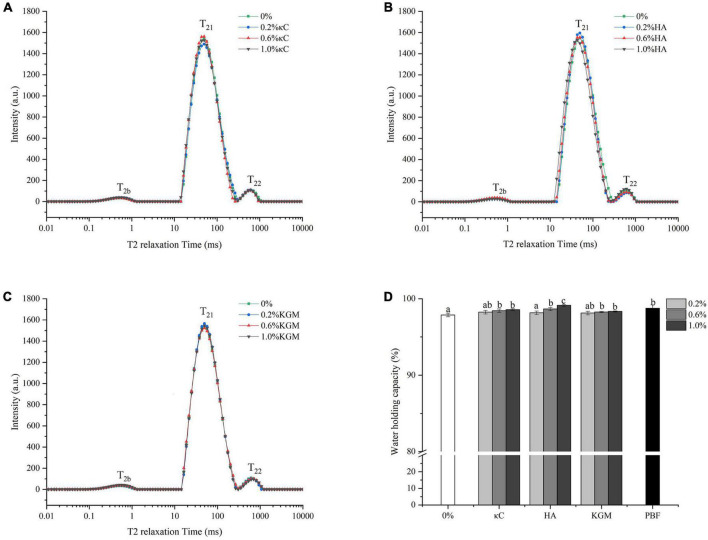
Water holding properties of emulsion gels containing different polysaccharides. **(A–C)** Transverse relaxation curves; **(D)** Water-holding capacity. Different letters indicate that the values are significantly different (*P* < 0.05). κC, κ-carrageenan; HA, high-acyl gellan; KGM, konjac glucomannanon; PBF, pig back fat.

Water molecules mobility and distribution in the gel systems were measured by LF-NMR. Water in gels can be divided into three types: T_2b_ was considered to be bound water, relaxation time mainly between 0 and 1 ms; T_21_ was considered to be immobilized water, which represented water trapped in the three-dimension network, and the relaxation time was mainly between 40 and 60 ms; T_22_ was assigned to be free water, relaxation time mainly between 600 and 800 ms (37). As shown in Figures 8A–C, the T_2b_ and T_21_ fractions accounted for more than 96% in all emulsion gels. Here, the T_21_ relaxation peak held an overwhelmingly dominant position, suggesting the water mobility remained highly restricted. The WHC of emulsion gels was shown in Figure 8D, which was consistent with the results of LF-NMR. Within the treatment concentrations (0.2–1.0% κC/HA/KGM), the WHC of PPI/polysaccharide emulsion gels slightly increased and reached a level comparable to pig back fat. It was reported that WHC was closely correlated with the density of the gel network structures (38, 39). In our work, the incorporation of polysaccharides formed a more heterogeneous microstructure (Figures 6, 7), suggesting that the WHC of the gel network structures was not be affected by the microphase separation of polysaccharide; while lower permeability of polysaccharides could allow water to be effectively “bound” in the emulsion gel matrices.

### FTIR spectroscopy

**FIGURE 9:**
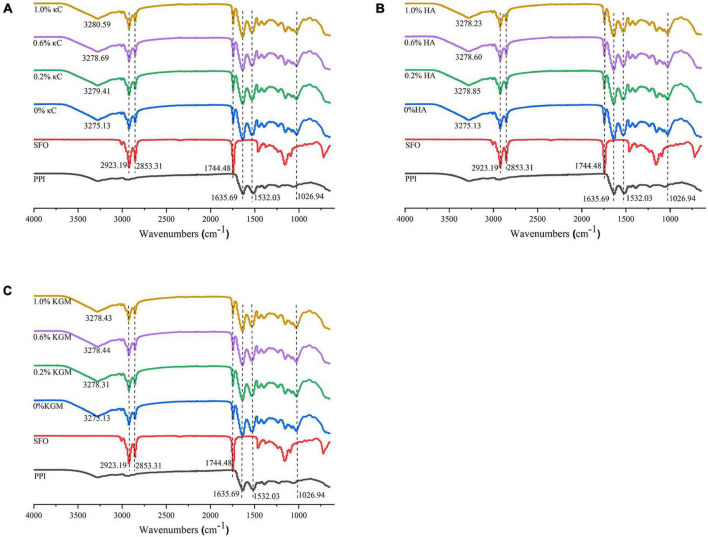
Infrared spectra of emulsion gels containing different polysaccharides: **(A)** κC, **(B)** HA, and **(C)** KGM. PPI, pea protein isolate; κC, κ-carrageenan; HA, high-acyl gellan; KGM, konjac glucomannanon; SFO, Sunflower seed oil.

The information of chemical interaction of the emulsion gels containing κC, HA, and KGM was further investigated by FTIR spectra. As shown in Figure 9, the broad absorption peak between 3,100 and 3,400 cm^–1^ was assigned to the response of hydrogen bond (O–H and N–H). With the addition of κC or HA or KGM, the peak of hydrogen bond moved to higher value, indicating the strength of hydrogen bonds was weakened in the emulsion gels during gel formation. It could be inferred that other forces were involved in the formation of PPI/polysaccharide emulsion gels. This observation agreed with the previous report of gelatin and κC/KGM composite gels (27). The strong absorption bands at 2,923, 2,853, 1,744, and 1,026 cm^–1^ belong to characteristic signals of sunflower seed oil. These results indicated that sunflower oil in the emulsion gels just as a filler, although the particle size of the oil droplets had changed.

Proteins had characteristic absorption bonds in the infrared region (40). The amide I band (1635.69 cm^–1^) was mainly caused by the C = O stretching vibration in the peptide bond. The amide II band (1532.03 cm^–1^) represented the bending vibration of the N-H group and the stretching vibration of the C-N group. For all PPI emulsion gels, increase in the absorption peak intensity at amide I and II were observed compared to PPI samples, which might be attributable to the formation of hydrogen bonds and cross-linking induced isopeptide bonds during the gel formation (41). Changes in secondary structures of the protein calculate from amide I were listed in Table 4. Overall, the α-helix content of emulsion gels after TG crosslinking decreased significantly, while β-sheet, β-turn and random coils slightly increased compared to uncrosslinked PPI, which might be due to the unfolding of protein during TG crosslinking process. The presence of different polysaccharides had little influence on the secondary structures, which further suggested their filler role in the emulsion gels.

**TABLE 4:** The composition of β-Sheet, random coils, α-Helix, β-Turns in secondary structure of emulsion gels containing different polysaccharides.

Sample	β -Sheet (%)	Random coils (%)	α -Helix (%)	β -Turns (%)
PPI	41.75 ± 0.57^a^	12.75 ± 0.06^b^	21.73 ± 0.20^a^	23.77 ± 0.25^c^
0% κC	42.44 ± 0.66^a^	13.94 ± 0.05^a^	14.18 ± 0.18^b^	29.40 ± 0.45^a^
0.2% κC	43.12 ± 0.27^a^	13.96 ± 0.09^a^	14.00 ± 0.10^b^	28.78 ± 0.22^ab^
0.6% κC	43.57 ± 0.18^a^	13.87 ± 0.03^a^	13.98 ± 0.02^b^	28.57 ± 0.18^b^
1.0% κC	43.13 ± 0.12^a^	13.91 ± 0.04^a^	14.05 ± 0.08^b^	28.93 ± 0.15^ab^
PPI	41.75 ± 0.57^a^	12.75 ± 0.06^b^	21.73 ± 0.20^a^	23.77 ± 0.25^c^
0% HA	42.44 ± 0.66^a^	13.94 ± 0.05^a^	14.18 ± 0.18^b^	29.40 ± 0.45^ab^
0.2% HA	43.18 ± 0.21^a^	13.96 ± 0.01^a^	14.05 ± 0.12^b^	28.81 ± 0.22^b^
0.6% HA	42.47 ± 0.29^a^	13.99 ± 0.05^a^	14.23 ± 0.15^b^	29.35 ± 0.19^ab^
1.0% HA	42.31 ± 0.05^a^	14.01 ± 0.06^a^	14.21 ± 0.11^b^	29.47 ± 0.08^a^
PPI	41.75 ± 0.57^a^	12.75 ± 0.06^b^	21.73 ± 0.20^a^	23.77 ± 0.25^c^
0% KGM	42.44 ± 0.66^a^	13.94 ± 0.05^a^	14.18 ± 0.18^b^	29.40 ± 0.45^ab^
0.2% KGM	43.08 ± 0.13^a^	13.92 ± 0.06^a^	14.10 ± 0.01^b^	28.93 ± 0.05^b^
0.6% KGM	42.30 ± 0.21^a^	13.94 ± 0.03^a^	14.21 ± 0.08^b^	29.52 ± 0.19^ab^
1.0% KGM	41.74 ± 0.14^a^	14.00 ± 0.05^a^	14.45 ± 0.09^b^	29.81 ± 0.07^a^

Different letters represent significant differences between different samples (*P* < 0.05).

PPI, pea protein isolate; κC, κ-carrageenan; HA, high-acyl gellan; KGM, konjac glucomannanon.

## Conclusion

In this work, the effects of polysaccharide type and concentration on the texture, rheological, microstructure and other functional properties of PPI/polysaccharide emulsion gels were studied, in order to explore the similarity to functional properties of pig back fat. The presence of polysaccharide significantly enhanced the hardness of all three PPI/polysaccharide emulsion gel systems, and PPI/κC system at a high polysaccharide content reached a similar level in hardness with pig back fat. Rheological results indicated that κC underwent gelation during protein gelation, which exhibited synergistic effects with PPI in term of improving the storage modulus (G’). The frequency sweep, the creep and recovery test results confirmed that the increase in polysaccharide concentration especially for PPI/κC system, had an obvious promoting effect on G’, instantaneous elastic behavior (G_0_) and delayed elastic behavior (G_1_), as well as the resistance against external forces. CLSM and SEM results indicated that the presence of polysaccharides destroyed the uniform distribution of protein network, resulted in the microphase separation (with different shapes of inclusions) as well as the broken and coalescence of oil droplets. However, the water holding capacity of PPI/polysaccharide emulsion gels was not affected by the microphase separation behavior. In the future, the plastic behavior and melting properties of PPI/polysaccharide emulsion gels will be further investigated to simulated more functional properties of pig back fat.

## Data availability statement

The original contributions presented in this study are included in the article/supplementary material, further inquiries can be directed to the corresponding author.

## Author contributions

WH: conceptualization, investigation, and writing – original draft. JL: data curation and investigation. YH: supervision and resources. YC: data curation and formal analysis. XK and CZ: writing – review and editing and validation. XL: methodology, conceptualization, and project administration. All authors contributed to the article and approved the submitted version.
